# ZSTK474, a specific class I phosphatidylinositol 3-kinase inhibitor, induces G1 arrest and autophagy in human breast cancer MCF-7 cells

**DOI:** 10.18632/oncotarget.7658

**Published:** 2016-02-24

**Authors:** Yaochen Wang, Jing Liu, Yuling Qiu, Meihua Jin, Xi Chen, Guanwei Fan, Ran Wang, Dexin Kong

**Affiliations:** ^1^ Tianjin Key Laboratory on Technologies Enabling Development of Clinical Therapeutics and Diagnostics, School of Pharmaceutical Sciences, Tianjin Medical University, Tianjin 300070, China; ^2^ Research Center of Basic Medical Sciences, Tianjin Medical University, Tianjin 300070, China; ^3^ State Key Laboratory of Modern Chinese Medicine, Tianjin University of Traditional Chinese Medicine, Nankai Dsitrict, Tianjin 300193, China

**Keywords:** ZSTK474, PI3K inhibitor, MCF-7, G1 arrest, autophagy

## Abstract

Multifaceted activities of class I phosphatidylinositol 3-kinase (PI3K) inhibitor ZSTK474 were investigated on human breast cancer cell MCF-7. ZSTK474 inhibited proliferation of MCF-7 cells potently. Flow cytometric analysis indicated that ZSTK474 induced cell cycle arrest at G1 phase, but no obvious apoptosis occurred. Western blot analysis suggested that blockade of PI3K/Akt/GSK-3β/cyclin D1/p-Rb pathway might contribute to the G1 arrest induced. Moreover, we demonstrated that ZSTK474 induced autophagy in MCF-7 cells by use of various assays including monodansylcadaverine (MDC) staining, transmission electron microscopy (TEM), tandem mRFP-GFP-LC3 fluorescence microscopy, and western blot detection of the autophagy protein markers of LC3B II, p62 and Atg 5. Inhibition of class I PI3K and the downstream mTOR might be involved in the autophagy-inducing effect. Combinational use of ZSTK474 and autophagy inhibitors enhanced cell viability, suggesting ZSTK474-induced autophagy might contribute to the antitumor activity. Our report supports the application of ZSTK474, which is being evaluated in clinical trials, for breast cancer therapy.

## INTRODUCTION

Breast cancer is the most frequently diagnosed cancer and the leading cause of cancer death among females worldwide, with an estimated 1.7 million cases and over 521 thousand deaths in 2012 [1]. Clinical treatment of breast cancer mainly includes surgery, radiation, and chemotherapy [2]. Side effects like lymphedema, numbness often occur after surgery and radiation, and current chemotherapy often leads to impaired fertility and premature osteoporosis, etc. [2]. Therefore, other chemotherapeutic agents with low side effects are still expected.

Class I phosphatidylinositol 3-kinases (PI3Ks), often referred to as PI3Ks, are lipid kinases that preferentially phosphorylate phosphatidylinositol 4,5-bisphosphate (PIP2) to generate phosphatidylinositol 3,4,5-trisphosphate (PIP3) which activates the downstream Akt and mammalian target of rapamycin (mTOR), and therefore stimulates signal pathways involved in cell proliferation, etc. [3]. The frequent amplification and gain-of-function mutations of PIK3CA which encodes PI3K catalytic subunit p110a, the dysregulation of PI3K/Akt pathway due to the upstream receptor tyrosine kinase (RTK), and the loss-of-function mutations of PTEN (phosphatase and tensin homolog deleted on chromosome 10), in cancer, suggest that PI3K is a desired target for cancer therapy [4]. GS-1101 (also called Idelalisib, CAL-101), a PI3K inhibitor specifically targeting PI3Kδ isoform [5] has been approved for chronic lymphocytic leukemia (CLL) therapy, while more PI3K inhibitors are under clinical evaluation. ZSTK474, 2- (2-difluoromethylbenzimidazol-1-yl) -4, 6-dimorpholino-1, 3, 5-triazine, is an orally administered pan-class I PI3K inhibitor identified by JFCR39 drug discovery system [6]. We previously reported that ZSTK474 exhibited potent antiproliferative effect on multiple cancer cell lines [7-9], antiangiogenic activity on human vein endothelial cells (HUVEC) [10] and antimetastatic activity on prostate cancer cell line PC3 [11]. PIK3CA mutation, HER2 amplification as well as PTEN mutation are frequently found in breast cancer [4, 12], suggesting that PI3K might be a promising target for breast cancer therapy. While the effect of PI3K inhibitors on cell cycle distribution and apoptosis were widely investigated, activity on autophagy was rarely reported. We previously reported the effects of 4 PI3K inhibitors (ZSTK474, BKM-120, NVP-BEZ235 and GDC-0941) on 14 cell lines including 3 breast cancer cell lines by using various assays [13]. While BKM-120 indicated more potent apoptosis-inducing activity compared with other 3 PI3K inhibitors, all of the 4 PI3K inhibitors generally showed autophagy-inducing activity, as evaluated by the ratio of LC3B II/LC3B I [13]. Autophagy has been recognized as a “double-edged sword” in cancer cells. The autophagy in response to cellular stress helps enhance cell survival by sustained energy production that can lead to tumor progression and drug resistance. However, excessive autophagy may promote cell death especially in apoptosis-defective cells [14], suggesting an approach for cancer therapy.

Therefore, we recently investigated the antitumor activities of ZSTK474 on breast cancer cell MCF-7, including those on cell cycle distribution, apoptosis, as well as autophagy.

## RESULTS

### ZSTK474 decreased MCF-7 cell viability

**Figure 1:**
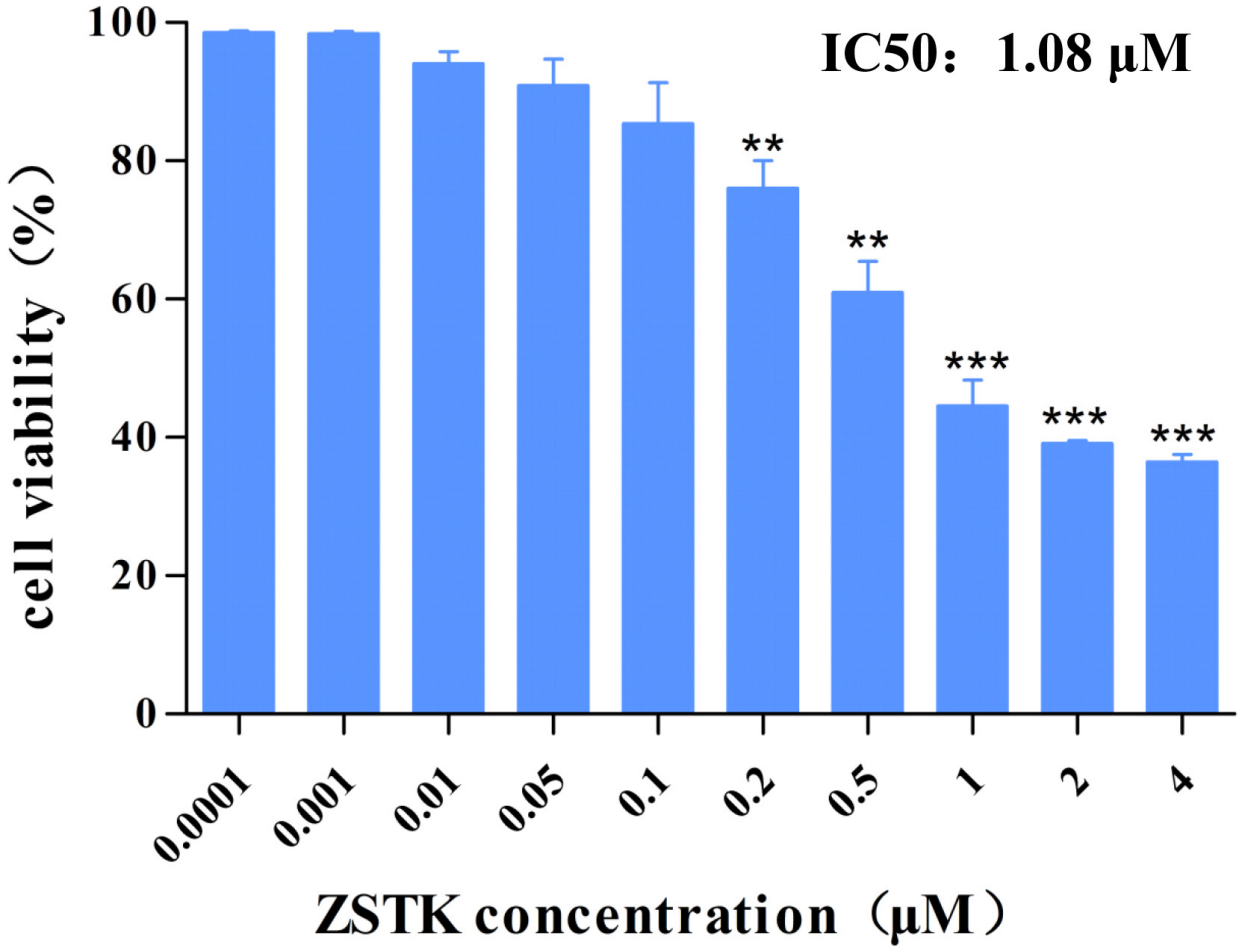
Effect of ZSTK474 on viability of MCF-7 cells MCF-7 cells were treated with various concentrations of ZSTK474 for 48 h. Number of viable cells was determined by MTT assay, and the cell viability (%) was calculated by comparison of ZSTK474-treated cells with control (without treatment). Data were presented as the mean ± S. D. (n=3). ** *p* < 0.01, *** *p* < 0.001, compared with control. ZSTK: ZSTK474.

To test the antiproliferative activity of ZSTK474 on MCF-7 cells, the cells were treated with various concentrations of ZSTK474 for 48 h. Number of viable cells was determined by MTT assay. As shown in Figure 1, MCF-7 cell proliferation was inhibited by ZSTK474 in a dose-dependent manner, with the IC50 as 1.08 μM.

### ZSTK474 induced G1 cell cycle arrest in MCF-7 cells

**Figure 2:**
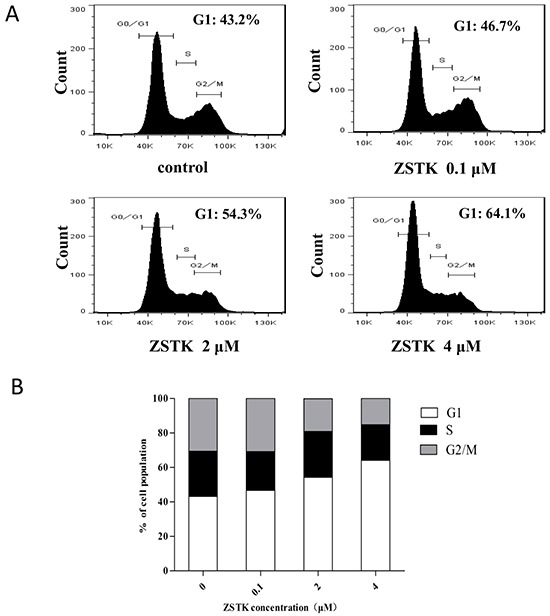
Effect of ZSTK474 on cell cycle distribution in MCF-7 cells **A.** MCF-7 cells were treated with various concentrations of ZSTK474 for 24 h. The cells were collected, dyed with propidium iodide, and analyzed by flow cytometer FACSVerse. **B.** Cell population (%) in each phase was analyzed by using Flow Jo Software. ZSTK: ZSTK474.

Since cell cycle progression is required for cell proliferation, we investigated the effect of ZSTK474 on cell cycle distribution in MCF7 cells. The cells were treated with 0, 0.1, 2, 4 μM of ZSTK474 for 24 h, stained with PI, and analyzed by flow cytometer. As a result, ZSTK474 induced G1 arrest in MCF-7 cells dose-dependently (Figure 2A, 2B). On the other hand, there was no sub-G1 population detected after treatment with ZSTK474, suggesting that this compound might not induce apoptosis in MCF-7 cells.

**Figure 3:**
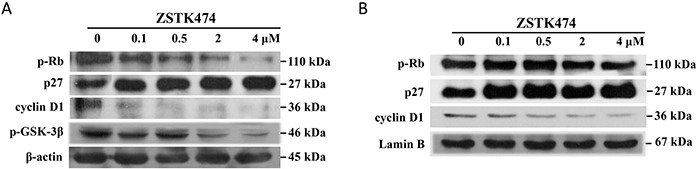
Effect of ZSTK474 on expression or phosphorylation of the cell cycle-related proteins in MCF-7 cells The cells were treated with 0, 0.1, 0.5, 2, 4 μM of ZSTK474 for 24 h. After treatment, the lysates of whole cell or nucleus were prepared by using the respective lysis buffer, to be available for western blot. The blots were exposed to anti- cyclin D1, p-GSK-3β, p27, phosphorylated p-Rb, β-actin (for whole cell) or Lamin B (for nucleus). Signals of the respective proteins in whole cell **A.** or nucleus **B.** after treatment with ZSTK474 were shown. Experiments were performed independently for three times.

Cell cycle progression is promoted by CDK (cyclin-dependent kinases)-cyclins, and inhibited by CDK inhibitors including p27. To investigate the mechanism for ZSTK474-induced G1 arrest, we examined the effect on the expression of cyclin D1, p27, as well as the downstream p-Rb by Western blot. As shown in Figure 3, after treatment with ZSTK474, either in total cell or in nucleus, the expression of p27 increased, while the levels of cyclin D1 and phosphorylated Rb decreased in a concentration-dependent manner, suggesting the inhibition against cyclin D1 expression and Rb phosphorylation, as well as increase of p27 expression, might be involved in ZSTK474-induced G1 arrest in MCF-7 cells.

It is known that cyclin D1 expression is mediated by GSK-3β, which is an effector downstream of PI3K/Akt signaling pathway [15]. To investigate whether the inhibition against cyclin D1 expression is related to the regulation of GSK-3β, we also determined the effect on GSK-3β expression. Figure 3A showed that the level of phosphorylated GSK-3β reduced dose-dependently after treatment, suggesting ZSTK474 inhibited the phosphorylation of GSK-3β probably via PI3K/Akt pathway.

### ZSTK474 did not induce apoptosis in MCF-7 cells

**Figure 4:**
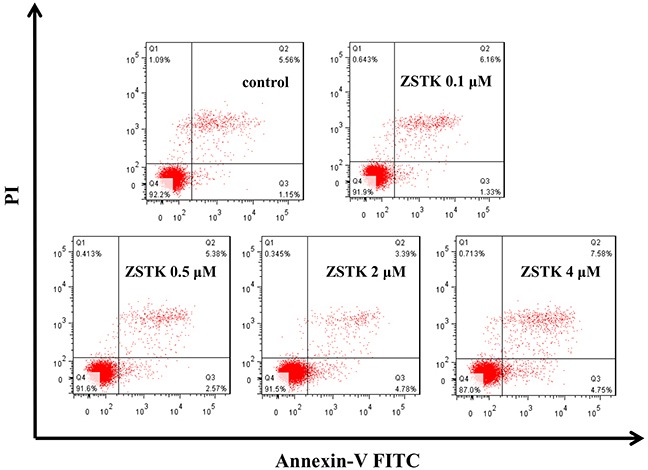
Effect of ZSTK474 on apoptosis in MCF-7 cells The cells were treated with 0, 0.1, 0.5, 2, 4 μM of ZSTK474 for 24 h. After treatment, the cells were collected, stained with Annexin V/PI, and analyzed by using flow cytometer FACSVerse. ZSTK: ZSTK474.

It is known that PI3K/Akt pathway activates to maintain cell survival. To investigate whether targeting PI3K by ZSTK474 inhibits the survival of MCF-7 cells, the apoptosis in MCF-7 cells after ZSTK474 treatment was determined by measuring phosphatidylserine (PS) externalization, which is known as a marker of apoptosis, with flow cytometer. As shown in Figure 4, compared with that in MCF-7 cells without treatment, no obvious increase of apoptotic cell population was detected in the ZSTK474 treated cells, indicating that ZSTK474 did not potently induce apoptosis in MCF-7 cells. This result is consistent with the data obtained from cell cycle analysis (Figure 2): no sub-G1 population detected in ZSTK474-treated cells.

### ZSTK474 induced autophagy in MCF-7 cells

Since autophagy is known to be inhibited by mTOR which is a downstream effector of PI3K/Akt pathway [16], pharmacological inhibition of PI3K might also induce autophagy. Then, we determined the effect of ZSTK474 on autophagy in MCF-7 cells by use of various assays. As a well-known mTOR inhibitor, rapamycin was reported to exhibit autophagy inducing activity [17] and therefore was used as a positive control in our experiments.

**Figure 5:**
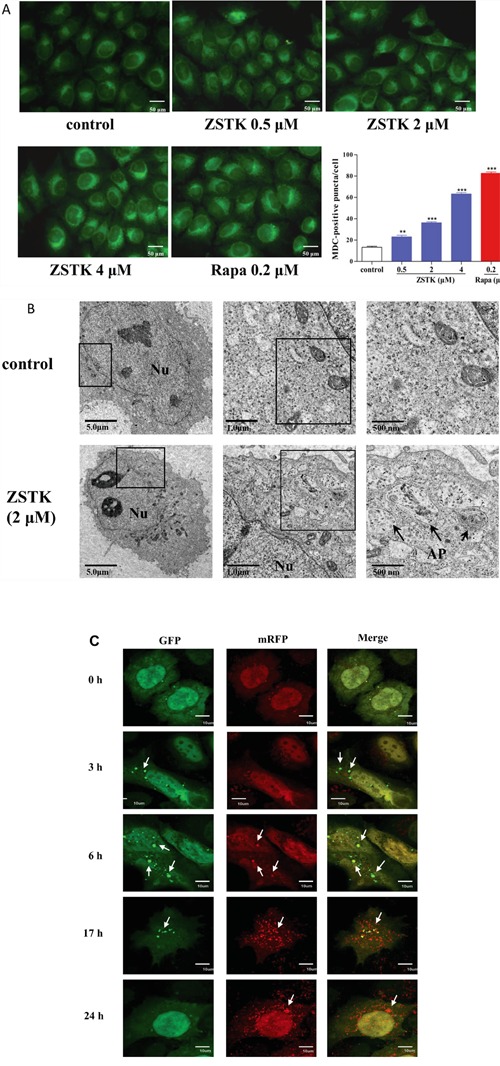
Effect of ZSTK474 on autophagy in MCF-7 cells **A.** MCF-7 cells were plated on coverslips in 6-well plates, treated with various concentrations of ZSTK474 (0, 0.1, 0.5, 2, 4 μM) or rapamycin (0.2 μM) for 6 h, then stained with 0.05 mM MDC for 1 h at 37°C. After washing with PBS, the stained MCF-7 cells were immediately examined by Olympus BX51 fluorescence microscope. Organelles stained with bright green fluorescence indicate the autophagic vacuoles. For quantification, MDC-positive puncta/cell in 20 cells chosen randomly was quantified using the Image J program. The results shown are mean ± SD; **: p < 0.01, ***: p < 0.001. **B.** MCF-7 cells were plated on 6-well plates, treated with DMSO (control) and 2 μM of ZSTK. Six hours later, the cells were harvested and fixed. The ultrathin 50 nm sections were cut, stained with 2% (w/v) uranyl acetate and lead citrate, then examined with an electron microscope Hitachi 600. Nu: nucleus; AP: autophagosome. **C.** MCF-7 cells were transfected for 5 h with a plasmid containing GFP/mRFP-tagged LC3 (tfLC3) reporter gene by use of lipofectamine 2000, then treated with 2 μM of ZSTK for 0, 3, 6, 17, and 24 h, respectively. The treated cells were fixed to be available for microscopy. The GFP/mRFP images were acquired using Olympus FV1000 laser scanning confocal microscope. Arrows indicate autophagosomes. ZSTK: ZSTK474. Rapa: rapamycin.

Firstly, monodansylcadaverine (MDC) incorporation assay was carried out. MDC is an autofluorescent substance which selectively accumulates in acidic vesicular organelles (AVOs), and therefore is used as a marker for autophagy. After staining with MDC, the MCF-7 cells treated with or without ZSTK474 were observed under fluorescence microscope. As shown in Figure 5A, the number of autophagic vacuoles increased in MCF-7 cells dose-dependently after treatment with ZSTK474, suggesting ZSTK474 induced autophagy in MCF-7 cells. As expected, rapamycin also increased the number of autophagic vacuoles.

Transmission electron microscopy (TEM) is known to be the most reliable method for monitoring autophagic morphology. Therefore, we used transmission electron microscopy to confirm the induction of autophagy by ZSTK474. As shown in Figure 5B, the double-membrane vesicles containing subcellular materials which represent formation of autophagosomes, were found in 2 μM ZSTK474-treated cells, but none in cells without treatment, demonstrating that ZSTK474 indeed induced autophagy in MCF-7 cells.

To evaluate the influence of ZSTK474 on the process of autophagosome formation, fusion with lysosome, and degradation, we transfected MCF-7 cells with a plasmid stably expressing GFP (green fluorescent protein) / mRFP (monomeric red fluorescent protein)-tagged LC3 (tfLC3), an autophagic flux reporter containing LC3 protein fused with GFP and mRFP [18]. At the early stage of autophagy, the tfLC3 localizes to autophagosomes, developing both mRFP (red) and GFP (green) signals. The GFP protein is acid sensitive and quenches quickly following fusion of autophagosomes with lysosomes due to the acidic environment, whereas mRFP is relatively stable in the acidic environment of the autolysosome [19]. Therefore, yellow (merge of green GFP signal and red mRFP signal) puncta indicate early autophagosomes while red puncta (red mRFP signal alone) represent late autolysosomes. As shown in Figure 5C, before treatment with ZSTK474, only weak signals of GFP and mRFP protein which represent diffuse LC3 protein were found in the cytoplasm. After treatment with ZSTK474 for 3 h, the mRFP and GFP signals began to accumulate, and reached to a peak at the time point of 6 h, suggesting the gradual increase of the autophagosomes in MCF-7 cells. At the time point of 17 h after treatment, the number of autophagosomes reduced compared with that at 6 h, suggesting the fusion with lysosomes. After treatment for 24 h, GFP signals were almost invisible, suggesting the late stage of autophagy. These data suggest that ZSTK474 induced autophagic vacuole formation at early time points and promoted fusion of autophagosomes at late time points.

**Figure 6:**
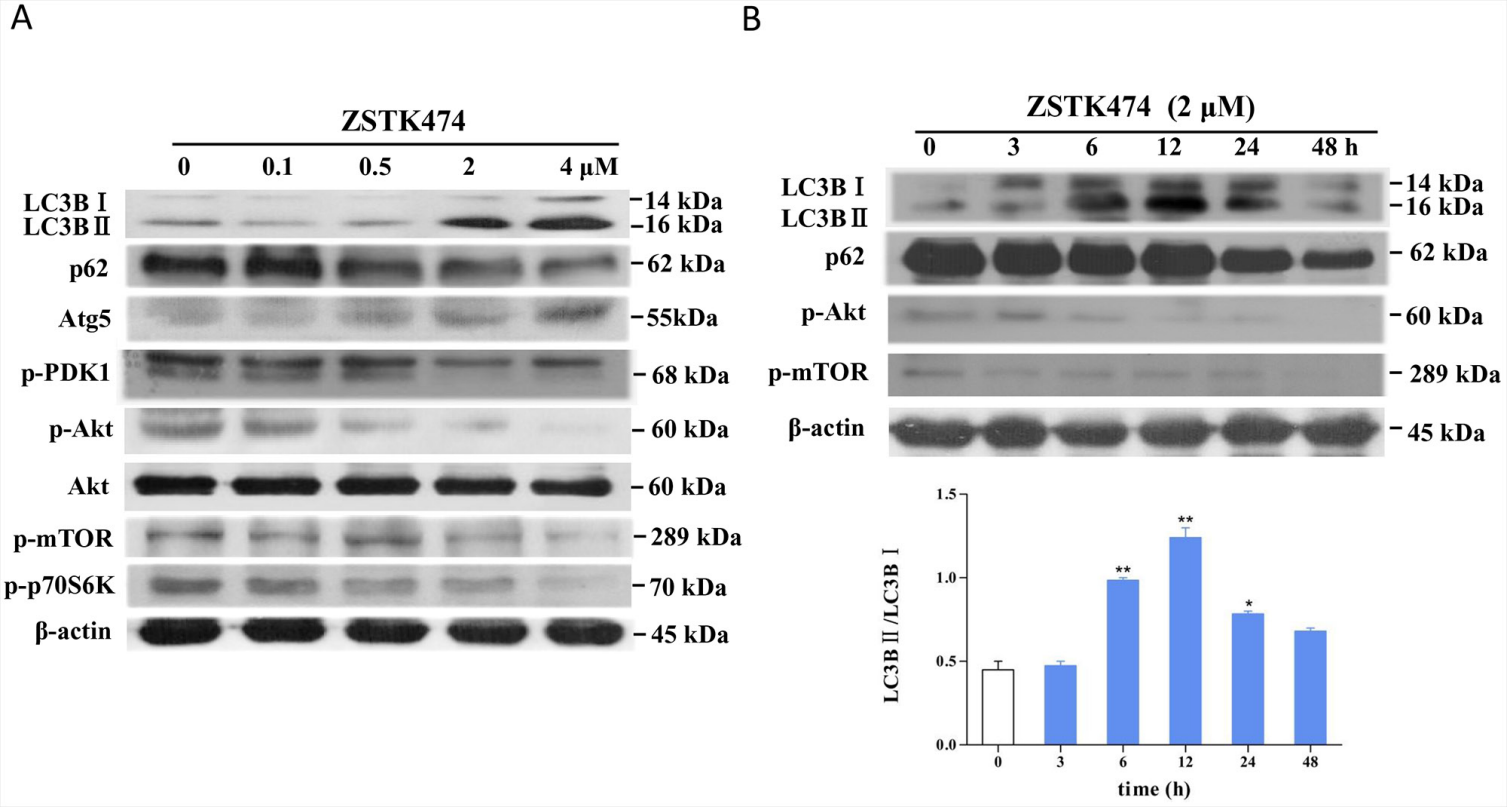
Effect of ZSTK474 on autophagy marker proteins as well as Akt/mTOR pathway signal proteins in MCF-7 cells **A.** Dose-dependent effect of ZSTK474 on autophagy marker proteins and the Akt/mTOR pathway signal proteins. The cells were treated with 0, 0.1, 0.5, 2, 4 μM of ZSTK474 for 6 h. Cell lysates were prepared to be available for western blot. The blots were exposed to anti- LC3B, p62, Atg5, p-PDK1, p-Akt, Akt, p-mTOR, p-p70S6K, and β-actin antibodies. And the bound signal proteins were further exposed to the respective secondary antibodies to be available for detection. **B.** Time dependent effect of ZSTK474 on autophagy marker proteins. The MCF-7 cells were treated with 2 μM of ZSTK474 for 0, 3, 6, 12, 24, 48 h, respectively. Cell lysate preparation and the western blot were performed as described in A, but the signals were exposed to anti- LC3B, p62, and β-actin (upper panel). The level of LC3B conversion (from cytosolic LC3B I to membrane-bound LC3B II) was calculated and shown in the lower panel of B.

Finally, we determined the effect of ZSTK474 on the expression of marker proteins of autophagy including LC3B II, p62 and Atg 5 by western blot analysis. As shown in Figure 6A, after treatment with ZSTK474 for 6 h, the conversion of LC3B I to II and the expression of Atg5 increased, while the expression of p62 decreased, further demonstrating the autophagy-inducing activity of ZSTK474. Interestingly, the conversion of the LC3B I to II seemed to increase gradually first, to attain the highest level at 12 h after treatment, and then to decrease until 48 h. (Figure 6B)

To investigate whether the autophagy-inducing activity of ZSTK474 is attributed to its class I PI3K inhibition, we also examined the effect of ZSTK474 on the downstream signal proteins of PI3K pathway. As indicated in Figure 6A, after treatment by ZSTK474 for 6 h, phosphorylation of PDK1, Akt, mTOR, as well as p70S6K was inhibited. Since mTOR is known to be a major gatekeeper to prevent autophagy in mammalian cells [20], the autophagy-inducing effect of ZSTK474 might be attributed to its inhibition against PI3K which activates the downstream mTOR.

### Pharmacological blockade of autophagy increases viability of the ZSTK474-treated MCF-7 cells

**Figure 7:**
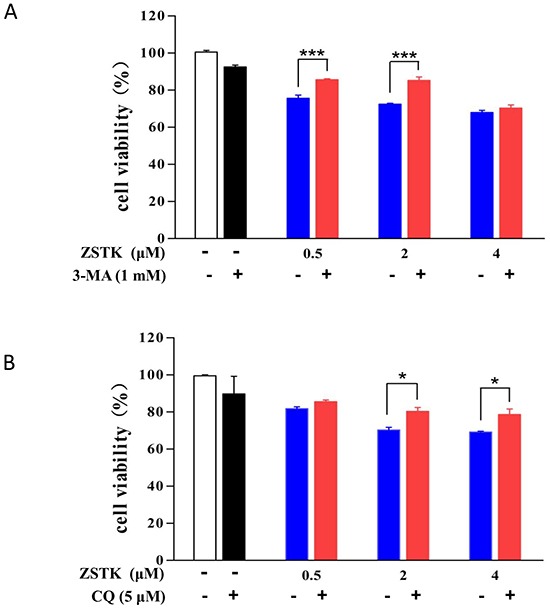
Blockade of autophagy increases viability of the ZSTK474-treated MCF-7 cells MCF-7 cells were incubated with or without autophagy inhibitor 3-MA (1 mM) **A.** or CQ (5 μM) **B.** for 1 h, followed by treatment with various concentrations of ZSTK474 (0, 0.5, 2, and 4 μM) for 24 h. Cell viability was determined by MTT assay and presented as the percentage of that for control (with neither ZSTK474 nor autophagy inhibitor treatment). Difference between ZSTK474 alone and ZSTK474 plus autophagy inhibitor was performed by using student's t test. * *p* < 0.05, *** *p* < 0.001. ZSTK: ZSTK474.

To investigate whether the autophagy-inducing activity of ZSTK474 would contribute to its antitumor activity or not, we first treated MCF-7 cells for 1 h with autophagy inhibitor 3-methyladenine (3-MA) or chloroquine (CQ), followed by exposure to ZSTK474 for 24 h. Then MTT assay was used to compare the cell viability for ZSTK474 alone with that for combination with autophagy inhibitor. As shown in Figure 7, co-treatment of either 3-MA or CQ led to the decreased anti-proliferative activity of ZSTK474, suggesting that the ZSTK474-induced autophagy contribute to its antitumor activity.

## DISCUSSION

ZSTK474 is a specific class I PI3K inhibitor we identified by using JFCR39 drug discovery system [21]. Although ZSTK474 has entered clinical trial, detailed study on specific type of cancer has not been reported. Since PIK3CA mutation was found in human breast cancer MCF-7 cells which might activate PI3K/Akt pathway, we recently investigated multifaceted activities of ZSTK474 on MCF-7 cells. ZSTK474 inhibited proliferation of MCF-7 cells with an IC50 as 1.08 μM. G1 arrest was induced, while no obvious apoptosis was observed in ZSTK474-treated MCF-7 cells. Moreover, ZSTK474 induced autophagy in MCF-7 cells, which might contribute to its antitumor effect.

Cell cycle progression is known to be promoted by cyclin-CDK complex and inhibited by CDK inhibitor proteins including p27. Treatment by ZSTK474 caused decreased expression of cyclin D1, but increased expression of p27, and the dephosphorylation of p-Rb. Since GSK-3β, a downstream effector of Akt, is reported to increase cyclin D1 expression [22], we then examined the level of phosphorylated GSK-3β after ZSTK474 treatment. As expected, the phosphorylation of GSK-3β was inhibited, suggesting the blockade of PI3K/Akt/GSK-3β/Cyclin D1/p-Rb signaling might be involved in the G1 arrest by ZSTK474. On the other hand, inactivation of Akt by ZSTK474 led to increase of p27 and the consequent desphosphorylation of p-Rb, which might also be involved in the G1 arrest.

Our result indicated that ZSTK474 did not induce obvious apoptosis in MCF-7 cells. While PI3K/Akt pathway is known to be essential for cell survival, most PI3K inhibitors including GDC-0941 and NVP-BEZ235 rarely induce apoptosis at their IC50 (concentration for 50% growth inhibition) concentrations [13]. Similar to our result, Dan S et al. [23, 24] also reported that ZSTK474 exhibited antitumor efficacy mainly via G1 arrest but not apoptosis induction. Moreover, MCF-7 cells were reported to be caspase-negative, which might be one cause for the none-induction of apoptosis.

Autophagy is an evolutionarily self-digesting process in which cytoplasmic material is sequestered within autophagosomes, and ended up in the lysosomal compartment. We utilized various assay methods to investigate the effect of ZSTK474 on autophagy. MDC staining and TEM showed the formation of autophagosomes. Tandem mRFP-GFP-LC3 fluorescence assay indicated the autophagy flux in MCF-7 cells after treatment by ZSTK474. Finally, we examined the effect of ZSTK474 on the expression of autophagy marker proteins including LC3B (also called Atg8) II, p62 and Atg5. LC3B II is known to bind with autophagosome, therefore conversion of LC3B I to LC3B II was considered to be a reliable marker of autophagy. Atg5 is involved in the two ubiquitin-like modification systems (the ATG12-ATG5 and the LC3 conjugation system) required during the formation of autophagosomes [25]. By linking ubiquitinated substrate with autophagic machinery, p62 is incorporated in completed autophagosomes and degraded in autolysosomes [17]. Treatment of MCF-7 cells with ZSTK474 for 6 h resulted in a cytoplasmic accumulation of LC3B II and Atg5 along with reduction of p62, further demonstrating that ZSTK474 induced autophagy in MCF-7 cells.

PI3Ks are a superfamily containing three classes, among which Class I and III PI3Ks are involved in autophagy [3]. Activation of the class I PI3K would inhibit autophagy via upregulating the downstream effector mTOR, which is known to negatively control autophagy [26]. In contrast, the class III PI3K Vps34 is required for the progression of autophagy [27]. As we reported previously, ZSTK474 showed potent inhibitory activity on 4 class I PI3K isoforms with IC50 values of less than 0.1 μM [28], while exhibited no inhibition against class III PI3K Vps34 even at 10 μM [9], suggesting its contribution to autophagy should be positive. Our present result showed that ZSTK474 treatment led to downregulation of p-Akt, p-mTOR as well as p70S6K, along with the increase of autophagy marker proteins like LC3B II, suggesting that ZSTK474-induced autophagy was attributed to its class I PI3K inhibition. This is the first detailed report about the autophagy-inducing effect of ZSTK474.

To investigate whether ZSTK474-induced autophagy would benefit its antitumor activity or not, we carried out two combination experiments by use of two autophagy inhibitors 3-MA and CQ. 3-MA is reported to inhibit autophagic sequestration while CQ acts to block the degradation of autolysosome [29]. 3-MA is an inhibitor of class III PI3K (Vps34) [30], which is required for autophagy progression [27]. Compared with use of ZSTK474 alone, combination with either autophagy inhibitor led to the decreased inhibitory activity on MCF-7 cell proliferation, suggesting the ZSTK474-induced autophagy might benefit its antitumor efficacy. In addition, autophagy is reported to promote secretion of ATP, which binds purinergic receptor P2RX7 in dendritic cells (DC), stimulates the recruitment of DCs into the tumor bed, and finally leads to the immunogenic cell death (ICD) of tumor cells [31, 32], further suggesting the potential contribution of ZSTK474-induced autophagy to the antitumor efficacy. The involvement of autophagic cell death in the in vivo antitumor activity would be investigated in the future.

MCF-7 was reported to be the first hormone-responsive breast cancer cell line which expresses estrogen receptor alpha (ERα) [33]. Mutations of PIK3CA are frequently (30%-40%) observed in ER positive breast cancers, suggesting the potential efficacy of PI3K inhibitors on such cancer cells [34]. In recent clinical trials, the efficacy of a pan-PI3K inhibitor BKM-120 in combination with ER down-regulator fulvestrant has been demonstrated [35]. While there are no established targeted agents for therapy of triple negative breast cancers (TNBCs) [36], one confirmed partial response (PR) was observed in a TNBC patient, after treatment with BKM120 [37], suggesting the potential efficacy of PI3K inhibitors on TNBCs.

In summary, ZSTK474 induced G1 arrest and autophagy, but no obvious apoptosis in human breast cancer MCF-7 cells. Induction of G1 arrest and autophagy might be attributed to the class I PI3K inhibition of this drug candidate. Besides MCF-7, we previously reported anti-proliferative activities of ZSTK474 on other breast cancer cells [21]. Moreover, oral administration of this compound at effective antitumor doses exhibited no obvious toxicity [10, 21]. Our report would support the promising therapeutic efficacy on breast cancer of ZSTK474, of which the clinical trial is ongoing in USA as well as in Japan.

## MATERIALS AND METHODS

### Reagents

ZSTK474 was purchased from Selleck (London, ON, Canada). Anti-p-Rb (S780), anti-p27, anti-Cyclin D1 antibodies were from BD Biosciences (San Jose, CA, USA), anti-phospho-GSK-3β (Ser9), anti-SQSTM1/p62 (D5E2), anti-Atg5, anti-LC3B, anti-phospho-Akt (Ser473), anti-Akt, anti-phospho-PDK1, anti-phospho-mTOR (Ser2448), anti-phospho-p70S6 Kinase (Thr389), and anti-β-actin antibodies were from Cell Signaling Technology (Danvers, MA, USA). Anti-Lamin B antibody was from Santa Cruz Biotechnology (Santa Cruz, CA, USA). The fluorescent dye monodansylcadaverine (MDC), Chloroquine diphosphate salt (CQ), 3-methyladenine (3-MA), and propidium iodide (PI) were purchased from Sigma-Aldrich (St. Louis, MO, USA). MTT (3-(4, 5-dimethyl-2-thiazolyl)-2, 5-diphenyl-2-H-tetrazolium bromide) reagent was from Amresco (Solon, OH, USA).

### Cell culture

Human epithelial breast cancer cell line MCF-7 was obtained from the American Type Culture Collection (ATCC) (Manassas, VA, USA) and grown according to the recommended guidelines. Cells were cultured in RPMI 1640 (Hyclone, Cramlington, UK) medium supplemented with 10% (v/v) fetal bovine serum (FBS) (Biological Industries, Kibbutz Beit-Haemek, Israel), 10 μg/ml streptomycin and 100 U/ml penicillin at 37°C in a humidified atmosphere containing 5% CO_2_.

### Cell viability assay

Cell viability was determined using MTT assay as described by us previously [38]. Briefly, following treatment of MCF-7 cells with various concentrations of ZSTK474 for 48 h in 96 well plates, MTT was added to each well at a final concentration of 0.5 mg/ml. To investigate the effect of autophagy on cell viability, cells were pre-treated with the 1 mM 3-MA or 5 μM CQ for 1h, then exposed to ZSTK474 for 24 h. After addition of MTT, the mixture was incubated for 4 h at 37°C. Then, the culture medium was removed, and the purple formazan crystal was dissolved with 150 μl DMSO. The resulting absorbance at 490 nm was determined by microplate reader (iMark, Bio Rad, Hercules, CA, USA). IC50 values were calculated by fitting the data points to a logistic curve using GraphPad Prism 4 Software (GraphPad Software, San Diego, CA, USA).

### Flow cytometric analysis

Cell cycle analysis was performed by flow cytometry after propidium iodide (PI) staining as we previously described [39]. MCF-7 cells were subcultured into 6-well plates at 37°C under a 5% CO_2_ atmosphere. Various concentrations of ZSTK474 (0, 0.1, 2, 4 μM) were added and further incubated for 24 h. Then, the cells were harvested, rinsed with cold PBS, and fixed with 75% cold ethanol solution at 4°C overnight. The cell suspension was centrifuged, and the resulting cell pellet was resuspended in 50 μg/ml of PI solution containing 0.5% Triton X-100 and 2% RNase A. The treated cells were incubated for 30 minutes in the dark at 4°C to be available for cell cycle analysis with flow cytometer (FACSVerse, Beckton Dickinson, Germany). Data were analyzed by using Flow Jo Software (Tristar, CA, USA).

Apoptosis analysis was carried out by detecting phosphatidyserine (PS) externalization using flow cytometry, as reported by us before [40, 41]. FITC Annexin V Apoptosis Detection Kit (BD Pharmingen, Oakville, ON, Canada) was used according to the manufacturer's protocol. After treated with various concentrations of ZSTK474 for 24 h, MCF-7 cells were harvested, washed with cold PBS, resuspended in 50 μl of annexin-binding buffer, and then incubated with 2.5 μl of FITC annexin V and 2.5 μl of PI for 15 minutes at room temperature in the dark. After dilution with 200 μl of annexin-binding buffer, the samples were available for flow cytometry analysis of apoptosis.

### Western blot analysis

Cell lysate preparation and western blot analysis were carried out as described previously with a small modification [10] As for detection of nuclear proteins such as p-Rb, p27, and cyclin D1, nucleus lysate was obtained using NE-PER Nuclear and Cytoplasmic Extraction Kit (Thermo Scientific, Rockford, IL, USA). Protein concentrations were determined with BCA Protein Assay Kit (Pierce, Rockford, USA). Equivalent amounts of protein were boiled in SDS sample-loading buffer, separated by sodium dodecyl sulfate polyacrylamide gel electrophoresis (SDS-PAGE) and transferred onto a PVDF membrane (Millipore, Billerica, MA, USA). The membranes were probed with the specified antibodies overnight and then hybridized with the respective HRP-conjugated secondary antibodies. Immunoreactivity was visualized using ECL detection reagents and digitalized by scanning. Final signals from the bound labeled- antibody was analyzed using Image J (National Institutes of Health, Bethesda, Maryland, USA).

### MDC incorporation

MDC staining was used to confirm the existence of autophagic vacuoles as reported previously [40]. Briefly, MCF-7 cells were plated on coverslips in 6-well plates (4×10^5^ cells/well) overnight at 37°C under a 5% CO_2_ atmosphere, then treated with various concentrations of ZSTK474 (0, 0.1, 0.5, 2, 4 μM) or rapamycin (0.2 μM) for 6 h, Cells were incubated with fresh medium containing 0.05 mM MDC for 1 h at 37°C. After washing with PBS, the stained MCF-7 cells on coverslips were immediately examined by fluorescence microscope (BX51, Olympus, Tokyo, Japan). For quantification, MDC-positive puncta/cell in 20 cells chosen randomly was calculated using the Image J program. The results shown are mean ± SD; **: p < 0.01, ***: p < 0.001.

### Transmission electron microscopy (TEM)

Morphological analysis utilizing electron microscopy was known to be the most reliable approach for monitoring autophagy [41]. MCF-7 cells were plated on 6-well plates (4×10^5^ cells/well) overnight at 37°C under a 5% CO_2_ atmosphere, then treated with DMSO (control) and 2 μM of ZSTK474, respectively. Six hours later, the cells were harvested and fixed. The ultrathin 50 nm sections were cut by use of an ultramicrotome, stained with 2% (w/v) uranyl acetate and lead citrate, then examined with electron microscope Hitachi 600 (Hitachi, Tokyo, Japan).

### Transfection assay and tandem mRFP-GFP-LC3 fluorescence microscopy

MCF-7 cells were transfected for 5 h with a plasmid (Addgene, Cambridge, MA, USA) containing mRFP-GFP-tagged LC3 (tfLC3) reporter gene which expresses LC3 protein fused with GFP and mRFP by use of Lipofectamine 2000 according to the instruction of the manufacturer. Then, the transfected (MCF-7-tfLC3) cells were seeded on glass coverslips in 24-well plates and incubated overnight. The next day, cells were washed and treated with ZSTK474 for 3, 6, 17, and 24 h, respectively. The treated cells were fixed for 15 min in 4% paraformaldehyde and washed with PBS. Finally, the coverslips were removed and mounted onto glass slides in Vectashield mounting media to be available for microscopy. The GFP/mRFP images were acquired using Olympus FV1000 laser scanning confocal microscope (Olympus, Tokyo, Japan).

### Statistical analysis

All of the experiments were performed in triplicate. Data were presented as means ± standard deviation (SD), representative of at least three independent experiments. Student's *t*-test was carried out for statistical significance analysis with GraphPad Prism (GraphPad, San Diego, CA, USA). Value of *P* < 0.05 was regarded as statistically significant.
